# De Novo Daptomycin-Nonsusceptible Enterococcal Infections

**DOI:** 10.3201/eid1804.110932

**Published:** 2012-04

**Authors:** Theodoros Kelesidis, Romney Humphries, Daniel Z. Uslan, David Pegues

**Affiliations:** David Geffen School of Medicine at UCLA, Los Angeles, California, USA

**Keywords:** Daptomycin, bacteria, enteric infections, Enterococcus, drug resistance, nonsusceptibility, antimicrobial resistance

## Abstract

Potential emergence of enterococcal daptomycin nonsusceptibility among patients with no prior exposure to daptomycin poses clinical and public health challenges. We found that development of infections with daptomycin-nonsusceptible enterococci in these patients could be associated with sporadic emergence and clonal spread.

The development of daptomycin resistance by enterococci poses treatment and infection control challenges. Emergence of daptomycin-nonsusceptible enterococci (DNSE) during treatment with daptomycin has been reported (*1*). We describe patient characteristics, clinical presentation, and outcome of 9 cases of DNSE infections in patients with no history of daptomycin treatment.

## The Study

We defined DNSE, by using the criteria of the Clinical and Laboratory Standards Institute (*2*), as enterococci with an MIC >4 ug/mL (*1*), as determined by in-house prepared reference broth microdilution (*3*) testing. We identified cases of DNSE infection by reviewing microbiology records from UCLA Health System during January 1, 2007–March 1, 2011. Patients with no history of daptomycin exposure and ≥1 positive clinical culture for DNSE were included in this study.

During the study period, we isolated 3,600 unique enterococci from adult inpatients at our facility and tested for antimicrobial drug susceptibility; 25 isolates were DNSE, 16 of which were recovered from patients who had received prior daptomycin therapy. We isolated DNSE from an additional 9 patients with no history of daptomycin use. Six (66.7%) patients were male and mean age was 58.9 years (range 26–75 years). Seven (77.8%) patients were immunosuppressed, of whom 5 had solid malignant tumors and 3 had diabetes. All patients in our case series had complicated concurrent medical conditions, and all but one had undergone surgery in the 3 months before isolation of DNSE (Table 1). For 4 patients, DNSE were isolated on the day of admission. For the remaining 5 patients, the average length of hospitalization before isolation of the first DNSE isolate was 45.4 days (Table 1).

**Table 1:** Patient and treatment characteristics for 9 patients with DNSE infection and colonization, Los Angeles, California, USA, 2007–2011*

Patient no., age, y/sex	Concurrent conditions†	Recent surgery	Site of isolation	*Enterococcus* species	Hospital day of DNSE isolation	Source of *Enterococcus* infection	Other pathogens isolated at DNSE site	Tx	Outcome of Enterococcus infection
1/69/M	RA on steroids, CHF, s/p AVR, s/p CABG	BKA, vascular surgery	Blood	*E. faecium*	47	CLABSI	None	DAP	Death
2/70/M	Diabetes, bowel disease, ILD on steroids, lung transplant	Thoracic	Blood, pleural fluid empyema	*E. faecium*	75	CLABSI, empyema	None	LZD, Q-D, GEN	Death
3/51/M	Colitis, TCC, severe hypocalcemia	No	Blood	*E. faecium*	17	CLABSI	None	VAN	Death
4/53/F	CNS tumor	No	Urine	*E. faecalis*	1	Asymptomatic bacteriuria	None	None	Undetermined
5/75/M	CVA	No	Urine	*E. faecalis*	1	UTI	None	LZD, VAN	Recovered
6/62/M	AIDS	Soft tissue	Wound	*E. faecium*	23	Chronic decubitus ulcerations	*E. coli*, MRSA, *Candida* spp.	LZD	Undetermined
7/64/F	Diabetes, bowel disease, splenectomy	GI tract	Abscess	*E. gallinarum*	1	Intra-abdominal abscess	Anaerobes, *S. viridans*	PIP/TAZ, ERT	Recovered
8/26/F	Cirrhosis, ESRD, breast cancer	GI tract	Abscess	*E. faecium*	65	Liver abscess	*E. coli, Klebsiella* spp., Stenotro.	LZD	Death
9/60/M	Diabetes, pancreatic cancer	No	Bile	*E. faecium*	1	Cholangitis	Fungi (*C. glabrata, A. fumigatus, S. cerevisiae*)	PIP/TAZ	Undetermined

*DNSE, daptomycin-nonsusceptible enterococcus; Tx, treatment; RA, rheumatoid arthritis; CHF, congestive heart failure; AVR, aortic valve replacement; CABG, coronary artery bypass graft; BKA, below knee amputation; CLABSI, central line–associated bloodstream infection; DAP, daptomycin; ILD, interstitial lung disease; LZD, linezolid; Q-D, quinupristin/dalfopristin; GEN, gentamicin; TCC, transitional cell carcinoma; VAN, vancomycin; CNS, central nervous system; CVA, cerebrovascular accident; UTI, urinary tract infection; *E. coli*, *Escherichia coli* ; MRSA, methicillin-resistant *Staphylococcus aureus*; GI, gastrointestinal; *S. viridans,* *Streptococcus viridans*; PIP/TAZ, piperacillin/tazobactam;ESRD, end-stage renal disease; Stenotro., Stenotrophomonas; *C. glabrata, Candida glabrata; A. fumigatus, Aspergillus fumigatus; S. cerevisiae, Saccharomyces cerevisiae* †Bowel disease was defined as presence of colitis, ischemic colitis, colonic ulcers, obstruction, or bowel surgery within 3 mo.

Use of antimicrobial drugs associated with presence of vancomycin-resistant enterococci, such as recent use of vancomycin, third-generation cephalosporins, or agents with activity against anaerobic bacteria (*4*), was associated with 5 (55.6%; mean duration 31.6 days, range 5–58 days), 1 (11.1%; duration 12 days), and 6 (66.7%; mean duration 33 days, range 5–65 days) patients, respectively. Recently, we suggested that the interplay between anaerobes and enterococci might have a possible role in the dissemination of daptomycin resistance (*5*). However, DNSE were also found in 3 (33.3%) patients (patients 3, 4, and 5; Table 1) who had no recent exposure to any antimicrobial agent. In addition, 2 patients (patients 4 and 5; Table 1) had no hospitalization or other health care exposure in the 12-month period before first isolation of DNSE, and DNSE were isolated on the first day of hospitalization, strongly suggesting community acquisition of DNSE.

Further support for a possible community reservoir of DNSE was provided by the identification of clonally related DNSE isolates. Of 9 DNSE isolates, 6 (66.7%) were *Enterococcus faecium*, 2 (22%) were *E. faecalis*, and 1 (11%) was *E. gallinarum*. Five isolates were available for further study (patients 4, 5, 6, 7, and 8) (Table 2). The daptomycin MICs were confirmed for these isolates by in-house prepared broth microdilution in cation-adjusted Muller Hinton broth plus calcium and by Etest (bioMérieux, Durham, NC, USA). Using strain typing by repPCR (DiversiLabTM; bioMérieux), we found that there was no genetic relatedness between the 2 *E. faecium* isolates available for typing, but the 2 *E. faecalis* were 97.7% similar by repPCR. These 2 isolates were also 98.5% related to a third daptomycin-nonsusceptible *E. faecalis* isolate from a patient who had received 90 days of daptomycin treatment before isolation of the DNSE. No epidemiologic link was found between these 3 patients, and 2 of the cases were identified on the first day of hospitalization, 6 months (patient 5) and 1 year (case 4) after the isolation of the original DNSE in the third patient (not included in this case series).

**Table 2:** Antimicrobial susceptibilities of DNSE isolates from 9 patients to antimicrobial drugs with activity against *Enterococcus* spp., Los Angeles, California, USA, 2007–2011*

Patient no.	*Enterococcus* species	MIC, μg/mL										Synergy test	
		DAP†	VAN	AMP	DOX	NIT	LZD	Q-D	TGC	CIP		GEN	STR
1	*E. faecium*	ND	>32 (R)	>64 (R)	<1 (S)	128 (R)	2 (S)	<0.5 (S)	<0.25 (S)	>4 (R)		S	S
2	*E. faecium*	ND	>32 (R)	>64 (R)	8 (I)	64 (I)	2 (S)	<0.5 (S)	<0.25 (S)	>4 (R)		R	S
3	*E. faecium*	ND	2 (S)	>64 (R)	16 (R)	32 (S)	1 (S)	<0.5 (S)	<0.25 (S)	>4 (R)		R	R
4	*E. faecalis*	4	1 (S)	<2 (S)	16 (R)	<16 (S)	2 (S)	4 (R)	<0.25 (S)	1 (S)		S	S
5	*E. faecalis*	12	2 (S)	<2 (S)	8 (I)	<16 (S)	2 (S)	4 (R)	<0.25 (S)	1 (S)		S	S
6	*E. faecium*	8	>32 (R)	>64 (R)	8 (I)	32 (S)	1 (S)	1(S)	<0.25 (S)	>4 (R)		S	S
7	*E. gallinarum*	4	<0.5 (S)	<2 (S)	<1(S)	32 (S)	1 (S)	1(S)	<0.25 (S)	<0.5 (S)		S	S
8	*E. faecium*	8	>32 (R)	>64 (R)	4 (S)	64 (I)	2 (S)	<0.5 (S)	<0.25 (S)	>4 (R)		R	S
9	*E. faecium*	ND	>32 (R)	>64 (R)	4 (S)	64 (I)	1 (S)	1 (S)	<0.25 (S)	>4 (R)		R	S

*DNSE, daptomycin-nonsusceptible enterococci; DAP, daptomycin; VAN, vancomycin; AMP, ampicillin; DOX, doxycycline; NIT, nitrofurantoin; LZD, linezolid; Q-D, quinupristin/dalfopristin; TGC, tigecycline; CIP, ciprofloxacin; GEN, gentamicin; STR, streptomycin; NS, nonsusceptible; ND, not done; R, resistant; S, sensitive. †By Etest.

Of the 9 patients, 8 showed evidence of clinical infection, including 3 bloodstream (33.3%), 3 intraabdominal (33.3%: 2 bile and 1 abscess), 1 urinary tract (22.2%), and 1 soft tissue (11.1%) infections. The remaining patient (patient 4) had asymptomatic bacteriuria with normal urinalysis. A potential nidus of infection, including a central venous catheter or inadequately drained abscess, was identified in 5 patients (55.6%) (Table 1).

Susceptibilities of the DNSE isolates to antimicrobial drugs with activity against the enterococci are summarized in Table 2. Fluoroquinolones, nitrofurantoin, doxycycline, and quinupristin/dalfopristin had activity against 3 (33.3%), 8 (88.9%), 7 (77.7%), and 7 (77.7%) of DNSE isolates, respectively. All isolates were susceptible in vitro to tigecycline and linezolid (Table 2). All but 1 *E. faecium* isolate were vancomycin resistant (83%, MIC >32 μg/mL) (Table 2), and all *E. faecalis* isolates were susceptible to vancomycin. As this series demonstrates, vancomycin-resistant enterococci and vancomycin-susceptible enterococci may be nonsusceptible to daptomycin. Although all laboratories are encouraged to investigate unusual MIC results by confirming organism identification and MIC result by the Clinical and Laboratory Standards Institute standards, the unusual MIC (e.g., daptomycin nonsusceptible) might or might not be relayed to the clinician in the instance of vancomycin-susceptible isolates.

Four (44.4%) patients died while receiving therapy for DNSE (Table 1). Each patient had multiple concurrent conditions, and cause of death could not be attributed solely to DNSE. (Table 1). Of the 5 patients who survived, the clinical response to treatment of DNSE infection could not be determined for 3 patients because of multiple concurrent conditions and polymicrobial infection. However, 1 patient with cholangitis, from whom DNSE were isolated from a bile culture, improved clinically despite receiving antimicrobial agents that were inactive against DNSE. The second clinically evaluable patient had asymptomatic bacteriuria associated with DNSE, did not receive any antimicrobial therapy, and remained clinically stable. Follow-up urine samples were cultured for 1 of the 5 surviving patients (patient 5); the result was negative.

## Conclusions

Two existing studies of de novo development of daptomycin nonsusceptibility describe 1 case each (*6**,**7*). To our knowledge, this study analyzes one of the largest series of DNSE isolates in patients with no prior exposure to daptomycin.

The mechanism for daptomycin nonsusceptibility in enterococci is poorly understood (*8*). A recent study found that 25% of *Enterococcus* spp. isolated from beef products were DNSE (*9*). Spread of these DNSE from agriculture to humans through the food chain may be a mechanism by which DNSE are emerging (*10**–**12*). Of note, in our case series, 3 patients (patients 2, 7, and 8) had a history of exposure to livestock: 1 (patient 2) was a veterinarian; 2 (patients 7 and 8) had histories of farm exposure. Three (patients 4, 5, and 7) reported frequent ingestion of beef. We recommend further investigation of these observations by case–control study.

The limitations of our study include the retrospective observational study design, the small number of cases identified, and lack of a comparison group. Case–control studies could better define risk factors associated with emergence of DNSE. Clinicians should be aware of the possibility of serious infections associated with DNSE even when there is no history of prior daptomycin therapy.
